# Rethinking MYC inhibition: a multi-dimensional approach to overcome cancer’s master regulator

**DOI:** 10.3389/fcell.2025.1601975

**Published:** 2025-04-29

**Authors:** Jing Yu, Dan Liu, Yujian Yuan, Chunxia Sun, Zihan Su

**Affiliations:** ^1^ Qingdao Hospital, University of Health and Rehabilitation Sciences, Qingdao, Municipal Hospital, Qingdao, China; ^2^ Qingdao Preschool Techers’ School, Qingdao, China

**Keywords:** MYC inhibition, cancer therapy, direct MYC targeting, indirect MYC targeting, combination therapies

## Abstract

MYC, a master regulator in oncogenesis, has long been deemed “undruggable” due to its intrinsically disordered structure. However, recent advances are overturning this view, with direct inhibitors like Omomyc (OMO-103) and PROTAC-based degraders such as WBC100 showing promising clinical progress. Complementary strategies—including BET and CDK9 inhibitors, RNA-based therapeutics, nanobodies, and engineered proteases—are expanding the therapeutic landscape. Despite challenges in specificity, toxicity, and delivery, these innovations underscore MYC’s emerging druggability. Moreover, combination therapies integrating MYC inhibitors with chemotherapy, radiotherapy, or immunotherapy demonstrate synergistic potential. This article advocates for a multi-dimensional, biomarker-guided approach to MYC targeting, emphasizing rational drug combinations and continued innovation to overcome resistance and improve outcomes in MYC-driven cancers.

## 1 Introduction

The MYC oncogene has been widely recognized as a master regulator implicated in numerous hallmarks of cancer, including uncontrolled proliferation, sustained tumor growth, metastasis, immune evasion, and therapy resistance (Papadimitropoulou et al., 2024; Whitfield and Soucek, 2025; Jha et al., 2023). Despite being among the most attractive therapeutic targets in oncology, MYC has historically been labeled as “undruggable” due to its intrinsically disordered structure, lacking defined binding sites required for traditional small-molecule inhibitors (Llombart and Mansour, 2022; Jain et al., 2024). This structural complexity, combined with concerns about the potential toxicity of MYC inhibition in normal proliferating cells, has significantly impeded the development of effective clinical therapies targeting MYC (Wu et al., 2023).

MYC overexpression or dysregulation has been implicated in a broad spectrum of malignancies, including breast, and ovarian cancers, among others (Zacarأas-Fluck et al., 2024; Bi et al., 2024). In breast cancer, MYC amplification is observed in approximately 15%–30% of cases, particularly in triple-negative breast cancer (TNBC) and HER2-positive subtypes, where it drives aggressive tumor behavior and resistance to standard therapies (Gao et al., 2023). In ovarian cancer, MYC plays a critical role in tumor progression by promoting metabolic reprogramming, chemoresistance, and invasion, making it a key factor in disease recurrence and poor survival rates (Liu et al., 2024; Yi et al., 2019). Beyond gynecological cancers, MYC is a major oncogenic driver in hematological malignancies such as Burkitt lymphoma, where its translocation leads to hyperactivation of proliferative pathways (Tandon et al., 2023). Additionally, MYC is frequently dysregulated in lung, colorectal, and pancreatic cancers, where its aberrant expression correlates with tumor aggressiveness and treatment resistance (Wallbillich and Lu, 2023; Dhanasekaran et al., 2022; Hessmann et al., 2016). These widespread implications underscore the urgent need for effective MYC-targeting strategies across multiple cancer types.

However, recent groundbreaking studies have begun to fundamentally reshape this perception. Advances in biotechnology and drug discovery, particularly the clinical success of the mini-protein inhibitor Omomyc (e.g., OMO-103) and emerging protein degradation strategies such as proteolysis-targeting chimeras (PROTACs), indicate that MYC inhibition is not only achievable but clinically viable (Garralda et al., 2024; Li et al., 2023). These innovative strategies are providing compelling evidence that MYC can be effectively targeted with acceptable safety profiles, opening an exciting new chapter in oncology. In this opinion article, we critically discuss recent progress and propose forward-looking approaches, advocating for a strategic and integrative framework to fully realize the clinical potential of MYC-targeted therapies.

## 2 Challenging the Myth of undruggable MYC

Although MYC has traditionally been viewed as ‘undruggable’ due to its structural features, recent biotechnological advances have directly challenged this notion. Innovative therapeutic modalities—including mini-proteins, proteolysis-targeting chimeras (PROTACs), and molecular glues—have demonstrated unprecedented potential to inhibit or degrade MYC with promising preclinical and clinical outcomes (Chen et al., 2021; Fred et al., 2015). Unlike conventional druggable targets such as kinases or enzymes, MYC lacks stable secondary and tertiary structures, making it difficult for small molecules to engage effectively. Traditional drug development approaches rely heavily on well-defined binding pockets, a feature conspicuously absent in the MYC protein. This structural challenge, compounded by MYC’s transient interactions with partner proteins such as MAX, previously led researchers to conclude that effective direct inhibition was impractical or even impossible (Zhao et al., 2024).

Nevertheless, emerging biotechnological advances and innovative therapeutic modalities have started to fundamentally challenge this assumption. Mini-proteins like Omomyc can disrupt MYC–MAX binding and block MYC’s DNA activity, offering new therapeutic potential (Al Masri and Yu, 2025). Concurrently, cutting-edge protein degradation technologies such as PROTACs and molecular glues offer novel means to specifically target and degrade MYC, circumventing the limitations posed by its disordered structure. These pioneering strategies not only provide direct evidence of MYC’s druggability but also highlight the significant progress being made toward overcoming historical barriers. Collectively, these advances argue compellingly that the categorization of MYC as “undruggable” is outdated, and justify renewed and intensified efforts to develop MYC-targeted therapies for clinical application.

However, a significant challenge in MYC-targeted therapies is the functional redundancy among MYC family members, including MYC, MYCN, and MYCL. These family members share considerable structural homology and often overlapping transcriptional targets, which can lead to compensatory mechanisms in cancer cells. Specifically, inhibition or degradation of one MYC protein may induce compensatory upregulation or activation of another, potentially diminishing therapeutic efficacy and fostering resistance. For example, in MYC-driven cancers, inhibition of c-MYC may trigger increased MYCN or MYCL expression, maintaining oncogenic signaling pathways. Addressing this functional redundancy will require combinational or pan-MYC strategies designed to simultaneously or sequentially inhibit multiple MYC family members, thereby enhancing treatment efficacy and minimizing resistance (Rickman et al., 2018; Carroll et al., 2018).

## 3 Direct MYC targeting and clinical progress

Direct targeting of MYC, once dismissed due to the protein’s intrinsically disordered nature, has witnessed remarkable progress through innovative therapeutic strategies, notably mini-proteins and protein-degradation approaches. Among the most promising developments is Omomyc, a groundbreaking 91-amino-acid mini-protein initially reported in 1998 as a proof-of-concept for effective MYC inhibition through homodimerization disruption (Soucek et al., 1998). Its clinical translation was significantly advanced by a pivotal 2019 study demonstrating intrinsic cell-penetrating properties, enabling its viability as a therapeutic candidate (Beaulieu et al., 2019). Omomyc specifically inhibits MYC by disrupting its heterodimerization with MAX and impeding DNA binding (Massأ٣-Vallأs and Soucek, 2020). Its clinically advanced derivative, OMO-103, recently achieved significant milestones, successfully completing a first-in-human Phase I clinical trial (NCT04808362) involving patients with various advanced solid tumors (Garralda et al., 2024). In this clinical trial, OMO-103 demonstrated not only a robust safety profile but also encouraging pharmacokinetics, effectively achieving tumor penetration and exhibiting preliminary evidence of clinical efficacy. Importantly, disease stabilization was observed in approximately half of the evaluable patients, with one metastatic pancreatic cancer patient experiencing an impressive 49% reduction in total tumor burden. These outcomes represent a pivotal breakthrough, definitively demonstrating the feasibility and potential efficacy of direct MYC inhibition in human cancers.

In addition to mini-protein inhibitors, recent advancements in targeted protein degradation using proteolysis-targeting chimeras (PROTACs) have provided another compelling avenue for directly targeting MYC. PROTAC technology overcomes traditional small molecule limitations by inducing targeted protein degradation, thus directly eliminating oncogenic MYC protein rather than merely inhibiting its activity. WBC100, a novel MYC-specific degrader, exemplifies this approach, and has progressed into Phase I clinical evaluation (NCT05100251) (Xu et al., 2022). Preclinical studies revealed that oral administration of WBC100 effectively reduced MYC protein levels in multiple tumor xenograft models, significantly inhibiting tumor growth and extending survival without overt toxicity. Such findings highlight the potential superiority of PROTAC-mediated degradation over conventional small-molecule inhibitors, primarily by addressing MYC’s rapid turnover and challenging structure.

These clinical advancements underscore the transformative potential of direct MYC targeting, marking a definitive departure from its historical characterization as “undruggable.” The clinical successes observed thus far, although preliminary, strongly support the continued development and clinical investigation of MYC-targeted therapeutics. These results warrant continued investigation of direct MYC inhibitors in diverse cancer contexts. Ultimately, the clinical progress achieved by mini-proteins such as OMO-103 and PROTAC degraders like WBC100 provides compelling evidence for direct MYC targeting as an emerging cornerstone in precision oncology.

## 4 Indirect approaches and their role in MYC inhibition

While direct MYC inhibition has garnered substantial attention, indirect strategies aimed at modulating upstream regulators or downstream effectors of MYC have also been extensively explored. Among these indirect approaches, bromodomain and extra-terminal (BET) inhibitors are perhaps the most prominent (Kong et al., 2022). BET proteins, particularly BRD4, are key transcriptional regulators that directly facilitate MYC gene transcription by binding to hyperacetylated chromatin regions near the MYC promoter (Lu et al., 2015). The BET inhibitor JQ1, along with analogs such as I-BET151 and OTX015, initially showed robust preclinical efficacy by dramatically downregulating MYC expression, resulting in significant tumor growth inhibition across diverse cancer models (Mertz et al., 2011). Despite promising preclinical data, however, clinical trials evaluating BET inhibitors have delivered mixed results. For instance, OTX015 (Birabresib) showed modest efficacy in hematological malignancies but encountered substantial toxicities including thrombocytopenia and gastrointestinal adverse events in Phase I/II clinical trials (NCT01713582). Moreover, the correlation between MYC suppression and clinical response to BET inhibitors was found inconsistent, as certain cancers exhibit BET inhibitor resistance despite elevated MYC expression levels (Berthon et al., 2016). These clinical experiences underscore that BET inhibitors, although promising, may not uniformly translate to MYC-specific clinical benefit, indicating a need for better patient selection and biomarker-guided clinical trials.

Another indirect strategy involves targeting cyclin-dependent kinase 9 (CDK9), which regulates transcription elongation of MYC-driven genes (Yan et al., 2024). CDK9 inhibitors such as KB-0742 have recently entered clinical trials (e.g., Phase I/II trial NCT04718675), with preliminary pharmacodynamic data showing effective reduction of MYC expression in preclinical cancer models. By blocking CDK9 activity, these inhibitors reduce transcriptional elongation and consequently suppress MYC and MYC-dependent oncogenic pathways (Villalona-Calero et al., 2024; Taghizadeh et al., 2024). Nevertheless, as transcriptional regulators, CDK9 inhibitors risk broad off-target effects due to the inhibition of global transcription processes, potentially limiting their therapeutic window (Borowczak et al., 2022). Clinical data from ongoing trials will further clarify whether CDK9 inhibitors can achieve selective therapeutic efficacy against MYC-dependent cancers without prohibitive toxicity.

Epigenetic modulation represents another important indirect avenue for MYC suppression. Omega Therapeutics’ OTX-2002, an innovative epigenomic controller delivered through lipid nanoparticles, targets and silences MYC expression pre-transcriptionally (Senapedis et al., 2024). Early-phase clinical studies (NCT05497453) have demonstrated encouraging preliminary safety and pharmacokinetic profiles, setting the stage for deeper clinical investigation (Mizrahi et al., 2025). While the specificity of epigenetic therapies remains an ongoing concern due to potential genome-wide effects, the ability of epigenetic modulators to sustainably repress MYC expression may offer long-lasting therapeutic benefits in selected MYC-driven tumors.

In summary, indirect MYC inhibition strategies provide valuable complementary approaches to direct targeting methods. While these approaches have not yet fully realized their initial promise in clinical trials due to inconsistent efficacy and considerable toxicity profiles, they remain critical components within a comprehensive therapeutic arsenal against MYC-dependent malignancies. Further refinement in patient selection and therapeutic specificity will be essential to realize the full potential of indirect MYC inhibition.

## 5 Emerging innovative strategies expanding MYC inhibition

In recent years, innovative therapeutic strategies have emerged that significantly expand the toolkit available for MYC inhibition, providing novel avenues to overcome the challenges previously associated with MYC targeting. Among these emerging strategies, antibody-based intracellular therapies represent a particularly exciting frontier (Wang et al., 2021). Typically, antibodies are limited to extracellular targets due to poor cellular penetration, but advanced antibody engineering techniques have now produced MYC-specific nanobodies capable of intracellular delivery. For example, recent preclinical studies demonstrated that anti-MYC nanobodies effectively penetrate cancer cells, selectively binding intracellular MYC protein and disrupting its transcriptional activity. This strategy directly inhibits MYC function at the protein level, reducing MYC-driven tumor growth *in vitro* and showing preliminary efficacy in xenograft mouse models (Lupanova et al., 2023). Although clinical translation remains at an early stage, these nanobodies hold considerable promise due to their specificity, low immunogenicity, and potential for high tumor penetration.

RNA-based methodologies represent another promising area for expanding MYC inhibition, particularly through RNA interference (RNAi) and RNA-degrading technologies. Traditional RNAi therapeutics targeting MYC, including small interfering RNAs (siRNAs) and short hairpin RNAs (shRNAs), previously encountered significant challenges related to stability, delivery, and off-target effects. However, breakthroughs in nanoparticle-based delivery systems have recently revitalized this approach. One recent study highlighted lipid-based nanoparticles delivering MYC-targeted siRNAs (such as DCR-MYC) into tumor cells, leading to efficient MYC silencing, potent anti-proliferative effects, and notable reduction of tumor burden in preclinical models (Raza et al., 2023). Additionally, newer RNA-targeting strategies such as molecular degraders that recruit cellular RNases specifically to MYC mRNA sequences have shown promising preclinical efficacy (Xie et al., 2018). Such precision RNA degradation techniques represent an elegant method of MYC modulation with potentially enhanced selectivity and minimal off-target effects compared to conventional RNA interference approaches.

Furthermore, innovative strategies employing engineered bacterial proteases have recently provided unexpected yet intriguing means for targeted MYC degradation. For instance, the bacterial Lon protease has been engineered for selective degradation of MYC protein in cancer cells (Butler et al., 2021; Ambite et al., 2025). Preclinical mouse models treated with recombinant Lon protease via intratumoral or systemic administration demonstrated significant MYC protein depletion, leading to reduced tumor growth and improved survival rates, particularly in bladder and colorectal cancer models. Although concerns remain regarding the immunogenicity and precise delivery of bacterial enzymes in human patients, this novel approach highlights the diverse potential inherent in biological degraders, offering entirely new modalities beyond traditional pharmacological compounds.

Collectively, these innovative emerging strategies—intracellular antibodies, RNA-based technologies, and engineered protein degraders—are expanding the boundaries of MYC inhibition research. Each of these novel approaches has demonstrated compelling preliminary data, underscoring their substantial therapeutic potential. While these strategies currently remain in preclinical or early clinical phases, continued investment, optimization of delivery systems, and rigorous validation in clinical settings will be crucial to fully realize their therapeutic promise. Ultimately, the integration of such advanced and diverse modalities into existing MYC-targeting paradigms could significantly enhance therapeutic outcomes, bringing MYC inhibition to the forefront of next-generation cancer therapies.

## 6 Strategic integration and combination therapies

MYC-driven cancers are complex. Combining MYC-targeted therapies with conventional treatments may enhance efficacy and reduce resistance. Direct MYC inhibitors, such as Omomyc-derived agents or PROTAC-based degraders, while promising, may encounter limitations as monotherapies due to adaptive resistance mechanisms inherent in cancer biology. Thus, combining these novel MYC-targeting agents with established therapies—including chemotherapy, targeted therapy, and immune checkpoint blockade—offers a compelling strategy to overcome such challenges.

Combination of MYC inhibitors with immune checkpoint inhibitors, for instance, represents an exciting and particularly promising therapeutic approach (Lee et al., 2022). MYC not only drives proliferation and survival pathways but also fosters an immunosuppressive tumor microenvironment through modulation of immune-related gene expression. Preclinical studies have shown that MYC inhibition significantly enhances tumor immunogenicity, increases T-cell infiltration, and potentiates immune checkpoint blockade efficacy. For example, combining Omomyc with anti-PD-1 therapy in mouse models has been shown to markedly improve response rates and survival outcomes compared to either treatment alone. This combinational synergy underscores the substantial potential for MYC inhibitors to serve as immune-modulatory agents, transforming poorly responsive tumors into immunologically active ones.

Furthermore, integrating MYC-targeted therapies with traditional chemotherapy or radiotherapy may also provide significant clinical benefits. Many chemotherapy agents, such as doxorubicin and paclitaxel, exert their effects partly through DNA damage-induced apoptosis, a pathway frequently counteracted by MYC-dependent survival mechanisms (Tang et al., 2022; Mekonnen et al., 2025). Recent preclinical data highlight that concurrent inhibition of MYC sensitizes cancer cells to chemotherapeutic agents, reducing chemotherapy doses required and potentially decreasing systemic toxicity. Similarly, preclinical studies have demonstrated that the combination of MYC inhibition with radiotherapy can significantly enhance tumor radiosensitivity and reduce radioresistance, suggesting potential improvements in local tumor control (Wang et al., 2022).

Another compelling approach involves simultaneous targeting of multiple MYC regulatory pathways. For example, combining direct MYC inhibitors with BET bromodomain inhibitors or CDK9 inhibitors might provide enhanced and sustained MYC suppression through complementary mechanisms (Zhang et al., 2023). This multi-modal targeting strategy could effectively limit the development of drug resistance, providing sustained therapeutic benefits. Although this approach requires rigorous preclinical validation and careful clinical optimization to manage overlapping toxicities, preliminary studies suggest strong potential for superior therapeutic outcomes compared to single-agent therapy.

Strategic integration and rational combination therapies represent essential future directions in MYC-targeted cancer treatment. The robust preclinical evidence supporting synergy between MYC inhibitors and various established therapies strongly advocates for carefully designed clinical trials incorporating these combinations. By strategically harnessing the complementary mechanisms of MYC-targeted agents alongside conventional or immune-based therapies, oncology research stands to substantially enhance treatment effectiveness, overcome adaptive resistance, and ultimately improve patient outcomes in MYC-driven cancers.

## 7 Conclusion and future perspectives

The longstanding perception of MYC as an “undruggable” target is rapidly being dismantled by groundbreaking advancements in drug discovery and therapeutic development. With the successful completion of the first-in-human clinical trial of OMO-103 and the emergence of novel protein degradation strategies such as PROTAC-based MYC degraders, direct MYC inhibition has transitioned from theoretical promise to clinical reality. These developments underscore a pivotal shift in cancer treatment paradigms, demonstrating that MYC inhibition is not only feasible but also potentially transformative in the management of MYC-driven malignancies. As summarized in Table 1, a variety of MYC-targeting strategies are currently at different stages of development, each with its own advantages and limitations, which will influence the future of these therapies. However, despite these advances, significant challenges remain, including optimization of drug delivery, identification of predictive biomarkers, and the need for robust strategies to mitigate resistance mechanisms.

**TABLE 1:** Overview of MYC-Targeting strategies and their clinical progress.

Strategy	Mechanism of action	Examples	Current status	Advantages	Limitations	Ref
Direct MYC Inhibition	Disrupts MYC-MAX dimerization, preventing DNA binding	Omomyc (OMO-103)	Phase I clinical trial completed (NCT04808362), showing disease stabilization in ∼50% of patients	First clinically validated direct MYC inhibitor, tumor penetration	Requires continuous administration, potential toxicity concerns	Garralda et al. (2024), Massأ٣-Vallأs and Soucek (2020)
PROTAC-Based MYC Degradation	Induces ubiquitin-proteasome degradation of MYC	WBC100	Phase I clinical trial ongoing (NCT05100251)	Selective MYC depletion, potential for high specificity	Delivery challenges, resistance mechanisms unknown	Li et al. (2023), Xu et al. (2022)
BET Bromodomain Inhibitors	Suppresses MYC transcription via BRD4 inhibition	JQ1, OTX015 (Birabresib)	Mixed results in Phase I/II trials (NCT01713582), limited efficacy in some cancers	Effective in MYC-overexpressing tumors	Dose-limiting toxicities (thrombocytopenia), resistance development	Kong et al. (2022), Lu et al. (2015), Mertz et al. (2011)
CDK9 Inhibitors	Blocks MYC transcriptional elongation	KB-0742	Phase I/II trial ongoing (NCT04718675)	Reduces MYC mRNA levels, synergistic with other therapies	Off-target effects, transcriptional toxicity	Villalona-Calero et al. (2024), Taghizadeh et al. (2024), Borowczak et al. (2022)
Epigenetic Modulation	Silences MYC expression pre-transcriptionally	OTX-2002 (epigenomic controller)	Phase I trial ongoing (NCT05497453)	Sustained MYC repression, potential long-lasting effects	Specificity concerns, need for biomarker-driven selection	Senapedis et al. (2024), Mizrahi et al. (2025)
RNA-Based MYC Inhibition	Targets MYC mRNA for degradation	DCR-MYC (siRNA-based therapy)	Preclinical and early-phase studies	Direct MYC suppression, potential for personalized therapy	Delivery limitations, stability issues	Raza et al. (2023), Xie et al. (2018)
Intracellular MYC-Targeting Nanobodies	Selectively binds intracellular MYC to disrupt function	MYC-specific nanobody constructs	Preclinical studies in xenografts	High specificity, immune evasion potential	Requires optimized intracellular delivery	Wang et al. (2021), Lupanova et al. (2023)
Bacterial Protease-Based MYC Degradation	Engineered proteases selectively degrade MYC	Lon protease therapy	Preclinical models show tumor suppression	Novel mechanism, non-genetic MYC depletion	Delivery challenges, immunogenicity concerns	Butler et al. (2021), Ambite et al. (2025)
Combination Therapies	Enhances MYC-targeting effects via synergy with other treatments	MYC inhibitors + PD-1 inhibitors	Preclinical and clinical combination trials	Synergistic anti-tumor activity, immune activation	Toxicity management, biomarker identification needed	Lee et al. (2022), Tang et al. (2022), Mekonnen et al. (2025)

Looking ahead, the future of MYC-targeted therapies will likely be shaped by three critical directions. First, continued innovation in drug development is essential to refine and expand the therapeutic arsenal against MYC. Emerging strategies such as intracellular MYC-targeting nanobodies, RNA-based MYC suppression, and engineered bacterial proteases represent promising alternatives that warrant further exploration. These strategies, together with refined mini-proteins and PROTACs, could enhance the precision and safety of MYC-targeted treatments while overcoming current limitations.

Second, a paradigm shift toward rational combination therapies will be crucial to maximize the clinical impact of MYC inhibition. Given MYC’s central role in oncogenic signaling and treatment resistance, combining MYC-targeted therapies with existing modalities—such as immune checkpoint inhibitors, chemotherapy, or radiotherapy—has the potential to generate synergistic effects and overcome intrinsic and acquired resistance. Future clinical trials should prioritize these combinational strategies, with a focus on optimizing treatment sequencing, dosing regimens, and biomarker-driven patient stratification to enhance therapeutic efficacy while minimizing toxicity.

Lastly, the development of robust biomarkers is imperative for guiding patient selection and optimizing MYC-targeted therapy. While MYC overexpression is a common feature across multiple cancer types, not all MYC-driven tumors are equally dependent on MYC activity for survival. Identifying reliable biomarkers—such as MYC transcriptional signatures, pathway activation markers, or novel imaging-based techniques—will be essential for selecting the right patient populations who are most likely to benefit from MYC inhibition. Integrating biomarker-driven approaches into future clinical trial designs will be critical for advancing personalized MYC-targeted therapies and improving overall treatment outcomes.

In conclusion, while Clinical trials have demonstrated measurable tumor response in directly and indirectly targeting MYC, further advancements in drug innovation, combination strategies, and biomarker development are necessary to fully harness the therapeutic potential of MYC inhibition. The next decade is poised to witness an accelerated evolution in MYC-targeted oncology, ultimately transforming this once “undruggable” oncogene into a clinically actionable therapeutic target. With sustained research efforts and strategic clinical implementation, MYC inhibitors hold the potential to reshape the future of cancer therapy, offering new hope for patients with MYC-driven malignancies.
